# Review of the clinical electrooculogram - Part 1: Mechanism of the Light-Rise

**DOI:** 10.1007/s10633-026-10103-z

**Published:** 2026-04-15

**Authors:** Paul A. Constable, Lynne Loh, Maja Šuštar Habjan, Srikanta Kumar Padhy

**Affiliations:** 1 College of Nursing and Health Sciences, Caring Futures Institute, Flinders University, Adelaide, Australia; 2 Department of Ophthalmology, University Medical Centre Ljubljana, Grabloviceva 46, 1000 Ljubljana, Slovenia; 3 Anant Bajaj Retina Institute, LV Prasad Eye Institute, Mithu Tulsi Chanrai Campus, Bhubaneswar, 751024 India

**Keywords:** Bestrophin, Light-peak, Retinal pigment epithelium, Anoctamin, Chloride, Calcium

## Abstract

The clinical electrooculogram (EOG) is the sole clinical electrophysiological test for assessing the function of the retinal pigment epithelium (RPE). However, despite several lines of investigation, the complete mechanism of the response has evaded a comprehensive description. The standard model implicates the rod photoreceptors and a signaling molecule termed the ‘light-rise substance’ that binds to an apical membrane ‘light-rise receptor’ or is transported across the membrane to elevate intracellular calcium concentration. The identity of the calcium activated chloride channel in the basolateral membrane was thought to be bestrophin, given the association of mutations in the hbest1 gene with Best Vitelliform Macular Dystrophy. However, recent findings have implicated a member of the anoctamin family as the calcium activated chloride channel with bestrophin regulating intracellular calcium in conjunction with the L-type calcium channel. How the changes in intracellular calcium are manifested as well as how the interaction with light in the dark-adapted state gives rise to the slow-dark and -light damped oscillations are yet to be described fully. This review summarizes the cellular mechanisms of the RPE that have been implicated in the generation of the light-rise and describes the likely candidates for the light-rise substance. A companion paper provides a summary of the bestrophinopathies and possible clinical modifications to enhance the EOG’s clinical utility.

## Background

The retinal pigment epithelium (RPE) is central to the origins of the clinical electro-oculogram (EOG) and this senescent cell is vital for the survival and maintenance of the outer retina throughout life. The RPE serves as a regulator of fluid in the sub-retinal space [1–3], phagocytosis of photoreceptor outer segments [4, 5], recycling of retinoids as part of the visual cycle [6], release of growth factors including pigment epithelial growth factor [7] and vascular endothelial growth factor [8] and supports potassium homeostasis in the sub-retinal space [9]. For a comprehensive review of RPE physiology see Strauss [10].

The clinical EOG has evaded a complete description of the mechanism since it was originally described by Arden and colleagues in the early 1960’s [11, 12]. The EOG assesses the functional integrity of the outer retina and RPE and plays a key role in the diagnosis of Best’s Vitelliform Macular Dystrophy (BVMD) [13]. The general model for the EOG is that the rods after a period of dark adaptation release a substance following light onset which binds to an RPE apical membrane receptor that leads to an increase in intracellular calcium concentration ([Ca^2+^]_in_) which gates open a basolateral chloride channel to depolarize the RPE’s basolateral membrane and initiate the light-rise of the EOG. The rationale for highlighting the rods in the mechanism of the light-rise was that the light peak amplitude depended on the duration of dark adaptation and presumably rhodopsin regeneration and that the spectral sensitivity of the light peak followed closely the absorption spectrum of rhodopsin [14]. The term ‘light rise’ refers to the increasing amplitude of the standing potential following light onset, whilst the ‘light peak’ is the maximal amplitude of the standing potential following light onset. For an earlier review of the EOG see Arden and Constable [15].

Central to our understanding of the changes in membrane potential across the RPE were original studies performed in RPE preparations to elucidate the ion channels, active transporters and cotransporters that regulate fluid transport, sub retinal ionic homeostasis and intracellular pH of the RPE [16–18]. However, the interactions with light and the photoreceptor-RPE complex are of interest to the generation of the clinical EOG and the fast oscillation (FO) that have different origins. The FO is typically recorded 10 repeated short intervals (~ 1 min) of dark and light in contrast to the clinical EOG with 15 min of dark and 15 min of light [19]. Their different origins are observed clinically in BVMD where the light-rise of the EOG is absent, but the FO remains unaffected [20]. The light-rise has its origins in the depolarization of the basal membrane due to increased chloride conductance of the RPE [2, 21, 22] while the FO are due to a delayed basolateral membrane hyperpolarization in response to a fall in sub-retinal potassium concentration following light onset [23].

Bestrophin-1, the product of *BEST1* (OMIM# 607,854) has been the focus of many groups since its classification as a calcium activated chloride channel (CaCC) [24] that was localized to the basal membrane using immunofluorescence in native porcine and macaque RPE [25]. However, when mouse knock-out models of *Best*^*−/−*^ the DC-ERG light-rise was not affected, which led to uncertainty about the role of bestrophin-1 in the light-rise [26]. The L-type Ca^2+^ was identified as an important voltage gated channel required for the light-rise when mice with mutations in the β subunit required a higher stimulus luminance than control mice to elicit an equivalent light-rise and nimodipine, an L-type Ca^+^ channel blocker, reduced the light-rise amplitude in control mice [26]. These findings identified [Ca^2+^]_in_ and the L-type Ca^2+^ channel as important in its generation. One further observation from this study [26] was that in the *Best*^*−/−*^ mice, when ATP was added to the apical membrane, to increases [Ca^2+^]_in_ there was a sustained (> 30 min) elevation in [Ca^2+^]_in_ which although not discussed at the time, was most likely due to a lack of function in bestrophin-1 as a regulator of stored Ca^2+^ in the endoplasmic reticulum [27].

Rosenthal et al. [28] identified an interaction between bestrophin-1 and L-type Ca^2+^channels in transfected RPE rat cell lines. Transfection with wild-type bestrophin-1 led to increased current kinetics with accelerated activation and a shift of the membrane potential to a more negative value. Yu et al. (2008) [29] found that bestrophin-1 binds via its C-terminus to the pore forming β-subunit of the L-type Ca^2+^ channel. The trafficking of bestrophin-1 into the plasma membrane of epithelial cell lines was reduced when the proline rich motif of the β-subunit was removed supporting a role for a close interaction between bestrophin-1 and the L-type Ca^2+^ channels that co-precipitated with bestrophin-1 [30]. The localization of bestrophin-1 to the basolateral membrane of porcine and macaque RPE provided further evidence for bestrophin being the CaCC responsible for the light-rise [25]. However, some doubts about the role and function of bestropin-1 remained given some discrepancies between animal and on the effects of bestrophin-1 on the light-rise being normal in knock-out mouse models [26] and in rat the overexpression of bestrophin-1 had a shifted luminance response of the light peak [31]. For review see Marmorstein et al. [32].

The identity of the basolateral CaCC is now presumed to be a member of the anoctamin family, and not bestrophin-1 as originally thought [30]. Anoctamin channels are expressed in epithelial and neuronal tissues and serve various functions including acting as CaCCs, scrambling phospholipids (moving the intracellular phospholipid membrane to the extracellular surface and vice-versa) [33]. In the RPE anoctamin-1 (a TMEM16A protein) is the likely candidate that depolarizes the basolateral membrane leading to the light-rise [34–36] with the light-rise substance released by the photoreceptors as possibly ATP or glutamate as proposed by Schreiber and Kunzelmann [36].

This review provides an overview of the models for the clinical EOG, based on evidence from human, animal and cell culture models. The review aims to build a case for considering the changes in the standing potential of the eye associated with the clinical EOG driven by ATP. The companion paper reviews the clinical applications in the bestrophinopathies and possible clinical modifications to reduce the testing time and improve patient comfort when recording the EOG [37].

## Retinal pigment epithelium ion channels and the standing potential

The RPE apical and basal membranes are separated by high resistance tight junctions that electrically couple two membranes and gives rise to the Trans Epithelial Potential (TEP). The TEP is defined as the difference between the basal and apical membrane potential; thus, changes in channel conductance in the apical or basal membrane will alter the recorded standing potential [38, 39]. The TEP of human adult RPE cells is ~ 3.5 mV with an apical membrane potential of approximately − 60 mV with the basal membrane potential being more hyperpolarized than the apical membrane. Thus, a depolarization of the basal or hyperpolarization of the apical membrane results in an increase in the TEP and standing potential whilst a hyperpolarization of the basal membrane or a depolarization of the apical membrane results in a decrease in the TEP and a fall in the standing potential of the eye. These membrane voltage changes are seen clinically as the light-rise of the EOG (basolateral depolarization) and the c-wave of the ERG (apical membrane hyperpolarization) [40]. Claudin-19 has been identified as a crucial component of maintaining the integrity and barrier function of the tight junctions [41]. Reductions in the amplitude of the recorded standing potential may arise in conditions that interfere with the barrier function of the tight junctions such as in Danon’s Disease where a normal LP:DT_ratio_ may be recorded in the presence of low absolute standing potential amplitudes [42]. Loss of barrier function that occurs in diabetes, will also reduce the c-wave, that is dependent on potassium currents in the RPE’s apical membrane [43]. For a review of the tight junctions in the RPE see Naylor et al. [44].

The RPE apical membrane contains the ubiquitous NaKATPase pump with a NaK2Cl co-transporter [45], voltage gated inward and outward rectifying potassium channels [46–48] that contribute to the regulation of potassium in the sub retinal space [17]. The sub-retinal potassium concentration is dependent on Na/KATPase pumps and delayed inward rectifying potassium channels in the inner segment [49] and the regulation of the cyclic nucleotide gated Na^+^/Ca^2+^ channel in the outer segment [50].The change in sub-retinal potassium concentration changes from ~ 5 mM to 2–3 mM during the transition from dark to light due to continued influx of potassium driven by the Na/KATPase pump [51]. A basolateral potassium channel is also present and contributes to the basolateral membrane potential [52]. The basal membrane also contains a slow L-type calcium channel [53–55] and fast T-type calcium channels [55]. Calcium is removed from the cytosol through active CaATPase pump [56] and the sodium-calcium exchanger [57]. The Transient Receptor Potential (TRP) channel also regulates calcium influx and may stabilize the basolateral membrane potential in conjunction with the sodium calcium exchanger [58]. Mathematical modelling of Ca^2+^waves in the ARPE-19 cell line have been proposed following mechanical stimulation and release of ATP to trigger an increase in [Ca^2+^]_in_ and its propagation with inositol tri-phosphate (IP_3_) to neighboring cells via gap junctions [59].

**Fig. 1:**
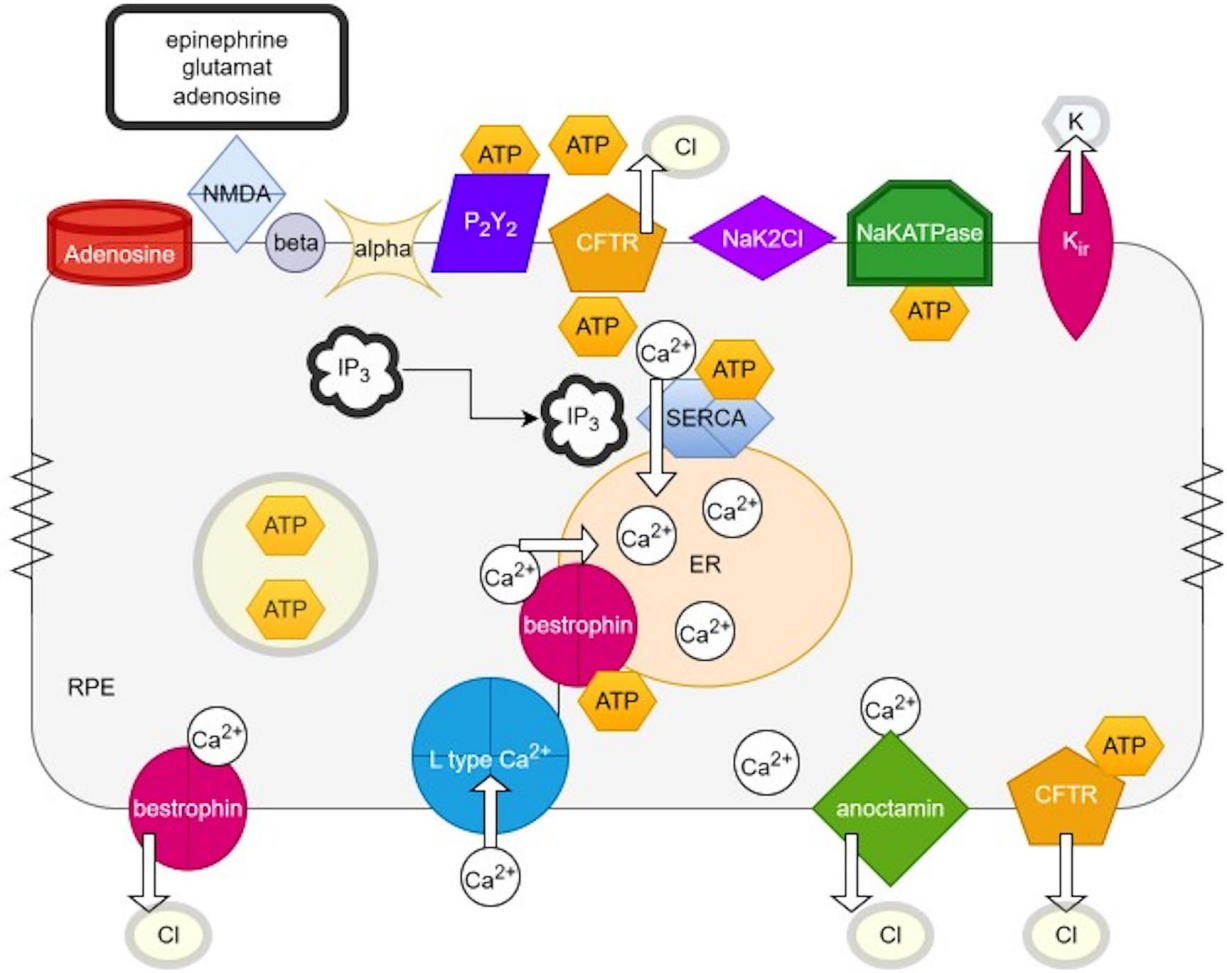
Outline of the key ionic channels and receptors in the retinal Pigment Epithelium apical and basal membranes related to the electrooculogram. At the apical membrane, there is the NaK2Cl co-transporter and active NaKATPase pump, with an inward rectifying voltage gated potassium channel. The apical cystic fibrosis transmembrane conductance regulator (CFTR) is gated by ATP at two nucleotide binding domains and transports chloride and ATP to the sub-retinal space. ATP may also be transported using vesicles to the apical membrane where alpha- and beta-adrenergic receptors are located. The purinergic (P_2_Y_2_), adenosine and glutamate NMDA receptors have also been identified in the apical membrane of the RPE. Within the cytosol the endoplasmic reticulum (ER) stores of [Ca^2+^]_in_ are maintained by the active endoplasmic reticulum calcium ATPase pump (ERCA) and bestrophin. Inositol triphosphate (IP_3_) binds to the IP_3_ receptor on the ER to release calcium and is formed following ligand binding with adrenergic receptors. Bestrophin is localized to the basolateral membrane and makes a link with the L-type Ca^2+^ channel that provides a pathway for calcium entry to the RPE. Bestrophin has a chloride conductance that acts as a counter ion for calcium uptake into the endoplasmic reticulum. Calcium when released from the ER gates opens the basolateral anoctamin channel which conducts chloride to depolarize the basolateral membrane and results in the light-rise of the EOG. CFTR is also present in the basolateral membrane of the RPE. Figure modified from Schreiber and Kunzelmann [36] and Mitchell and Reigada [71]

Several chloride channels are also present in the RPE with their main function in fluid regulation [3, 60]. The chloride channels include the cyclic adenosine monophosphate gated cystic fibrosis transmembrane conductance regulator (CFTR), which along with chloride ion transport facilitates ATP release from the RPE’s apical membrane via vesicular transport [61] to stimulate apical purinergic receptors and elevate [Ca^2+^]_in_ [62]. The apical membrane chloride channel protein 2 (CLC-2) is postulated to provide a role in chloride ion homeostasis in the sub-retinal space with knock-out mice developing outer retinal degeneration [63]. The Chloride Intracellular Channel (CLIC-4) [64] is localized in the RPE’s apical micro-villi and is critical for RPE cell communication with the outer segments [65] and extracellular matrix remodeling [66]. Patch clamp experiments in mouse RPE apical and basal membranes detected a chloride transporter with high permeability for thiocyanate and chloride [67]. The solute carrier family 27 member 7 (SLC26A7) was identified as the thiocyanate anion exchanger in the basolateral membrane of mouse RPE cells where it contributes to anion transport and pH regulation through Cl^−^/HCO_3_^−^ exchange [68]. Adrenergic [69] and purinergic receptors [70, 71] are present in the apical membrane of the RPE providing pathways for modulation of [Ca^2+^]_in_ and cyclic AMP as intracellular second messengers [69, 72–75]. NMDA receptors that bind glutamate to increase ATP release from bovine RPE preparations have also been identified providing a potential pathway for light mediated modulation of [Ca^2+^]_in_ with glutamate concentration increasing in dark and decreasing in light the direct involvement of glutamate is counter-intuitive to the light-rise [76]. Figure 1 summarizes the ion channels and receptors identified in the RPE.

## Bestrophin

The ‘classic’ condition in which the light-rise is absent is BVMD [20] that is the result of mutations in *BEST1* and thus bestrophin’s role in the RPE is central to the generation of the light-rise [77–80]. However, the function of bestrophin was not clear as it was presumed to be the basolateral CaCC, but its actual function is more complex in the generation of the light-rise and has taken time to piece together with several previous reviews on bestrophin describing its structure and function. See Johnson et al. [81], Guziewicz et al. [82], Pasquay et al. [83], Xiao et al. [13], Marmorstein et al. [32] and Hartzell et al. [84]. The locus for human bestrophin (*BEST1*) MIM# 607,854 was identified on chromosome 11q13 in a family with Best Macular Dystrophy (BMD) [85]. The gene that encodes the bestrophin protein originally described as vitelliform macular dystrophy VMD2 contained 11 exons that encodes a 585 amino acid protein with a mass of 68 kD and is strongly expressed in the RPE [85]. For reviews of the genetics of *VMD2/BEST1* see Nowomiesjska et al. [86], White et al. [87] and the companion paper.

## Bestrophin and the L-type calcium channel

Animal studies identified calcium signaling in the pathophysiology of BVMD in mouse models [88]. Blockade of L-type Ca^2+^ channels with nifedipine in human also reduced the amplitude of the light-rise [89] and similar effects were described with nimodipine in rodent models [26, 28]. Bestrophin was originally thought to be the basolateral CaCC [25, 90], but its role was postulated to be more complex and more as a regulator of [Ca^2+^]_in_ and chloride conductance in epithelia [90]. Interactions between bestrophin and the L-type Ca^2+^ were identified by Rosenthal et al. [28] who found that rats transfected with *BEST1* mutants modified the kinetics and voltage gating of the channel. The interaction was between the beta-subunit of the L-type Ca^2+^channel [29] and the src-homology binding domain of bestrophin [29]. Direct interaction between bestrophin-1 and the β subunit of the L-type-Ca^2+^ channel were confirmed through co-immunoprecipitation analysis in epithelial cell lines [91]. Bestrophin also supports the expression and localization of L-type-Ca^2+^ channels [92] further implicating a close relationship between bestrophin and the L-type Ca^2+^ channel.

## Bestrophin and calcium regulation

The path to unraveling the functional association between Ca^2+^ signaling and basolateral chloride conductance began with the discovery that bestrophin was also expressed in the endoplasmic reticulum of cell lines where purinergic agonists increased store operated release of [Ca^2+^]_in_ [93]. With the understanding that bestrophin was not exclusively expressed in the RPE basolateral membrane led to the implication for the crucial role of bestrophin to play in regulating release of Ca^2+^ from intracellular stores. Phosphorylation of bestrophin-1 by Pak2 (p21-activated kinase 2) enhanced the outward ATP induced CaCC currents in cell lines and co-located with bestrophin-1 [93]. In addition, Pak2 also activated the calcium dependent potassium channel (SK4) potassium channel providing a mechanism to regulate basolateral membrane voltage. The observation that Ca^2+^ deposits and expansion of the endoplasmic reticulum in airway epithelial cells of mBest1 knock-out mice further implicated bestrophin in [Ca^2+^]_in_ regulation [93]. In primary cultures of wild type and mBest1^−/−^ mouse RPE cells, the role of bestrophin-1 on calcium homeostasis was further explored by Neussert et al. [94] who found that at rest the [Ca^2+^]_in_ was higher in the knock out mouse cultures implying a role for Best1 in maintaining intracellular Ca^2+^stores and potentially acting as a chloride transporter as a counter-ion to facilitate Ca^2+^ entry into the endoplasmic reticulum. Co-localization of bestrophin-1 was primarily identified in cellular fractions from porcine RPE cells in the endoplasmic reticulum with less localization in the basolateral membrane fraction [94] indicating that bestrophin was not simply the basolateral CaCC as originally thought [25].

The mechanism of bestrophin mediated regulation of stored calcium was described by Cordeiro and Strauss [95] who used RT-PCR to demonstrate the expression of orai-1, 2 and 3 channel proteins and their stimulators, stim-1 and stim-2 in freshly isolated human RPE cells and a human RPE cell line (ARPE-19). Central to the regulation of store operated calcium channels in the endoplasmic reticulum are Stim-1 and Stim-2 that sense the depletion of calcium in the endoplasmic reticulum when it is released and orai calcium channels are activated by stim-1 and stim-2 to open and replenish cytosolic calcium levels for re-uptake into the endoplasmic reticulum. For review of stim/orai channels see Fahrner et al. [96]. Gómez et al. [27] found that bestrophin-1 had a 2.5 fold higher level of expression in the endoplasmic reticulum than the membrane in mice RPE cells. Furthermore, the store operated calcium entry was reduced in the presence of the orai-1 channel inhibitor, 2-aminoethoxydiphenyl borate, and by siRNA knockdown of orai-1 expression, confirming the role of orai-1 in regulating calcium re-uptake in RPE cells. Similarly, siRNA knockdown of bestrophin-1, in isolation, also strongly reduced [Ca^2+^]_in_ supporting an interaction between bestrophin-1 and orai-1 in calcium re-uptake [27].

**Fig. 2:**
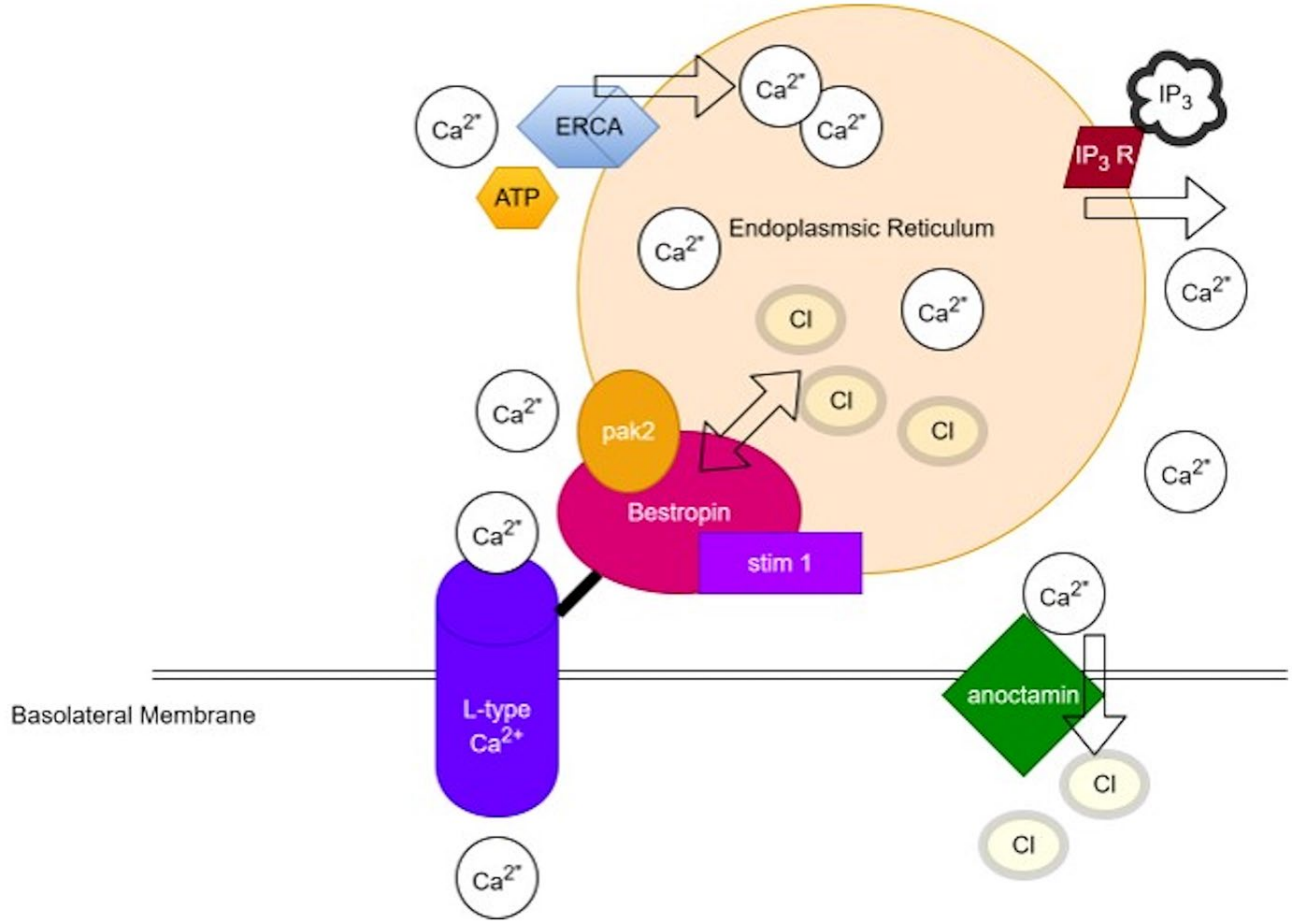
Summary of the interactions of Bestrophin and the regulation of intracellular calcium in the Retinal Pigment Epithelium (RPE). Bestrophin is mainly localized to the endoplasmic reticulum. Bestrophin is phosphorylated by ATP and p21 activated kinase (pak21) to increase chloride conductance into the endoplasmic reticulum that acts as a counter ion to facilitate calcium re-uptake into the endoplasmic reticulum. Stim sensors stored calcium concentration to regulate the activity of bestrophin binds to the basolateral L type Ca^2+^ channel to facilitate calcium entry to the cytosol. Calcium is taken up into the endoplasmic reticulum by the endoplasmic reticulum calcium ATPase pump (ERCA). Release of calcium is by inositol triphosphate (IP_3_) phosphorylation of IP_3_ receptors on the endoplasmic reticulum which releases calcium. The basolateral calcium gated TMEM16 family member anocamin-1 is gated by calcium to increase basolateral chloride conductance. Figure adapted by from Barro-Soria et al. [93]

In summary, bestrophin-1 is predominantly expressed in the endoplasmic reticulum where it regulates Ca^2+^re-entry into the endoplasmic reticulum following depletion through colocalizing with the calcium sensing protein stim-1 and through direct interactions with the L-type-Ca^2+^ channel and orai proteins to replenish cytosolic calcium. Bestrophin-1 also has a chloride conductance that acts as a counter ion to further increase the driving force for calcium reuptake into the endoplasmic reticulum. Through TMEM16A and SK4 CaCC and calcium activated potassium channels the basolateral membrane potential can be regulated and through membrane depolarization activation of the L-type Ca^2+^ channel opens for the entry of calcium into the cytosol for reuptake into the endoplasmic reticulum via the sarcoplasmic reticulum Ca-ATPase pumps. Figure 2 provides an overview of the interactions of bestrophin with calcium, chloride and potassium currents.

## The Basolateral calcium activated chloride channel

The basolateral CaCC identified in RPE cells belongs to the trans membrane (TMEM)/anoctamin family with TMEM protein16A (TMEM16A) members exhibiting CaCC properties [97]. The term anoctamin relates to their anion permeability and eight transmembrane loops [98]. Anoctamin-1 is a calcium and voltage gated ion channel that can be gated either by calcium, membrane voltage or by interactions between calcium and membrane voltage [99]. At the cytoplasmic facing lipid membrane phosphatidylinositol 4,5-bisphosphate (PIP2) stabilizes the current and regulates anoctamin-1 function [100]. In addition, the CaCC regulator 1 (CLCA1) colocalizes, upregulates and stabilizes the expression of anoctamin-1 in transfected human cell lines [101] and CLCA1 is expressed in canine RPE and increases Ca^2+^dependent chloride currents [102]. For a review on the anoctamins see Schreiber et al. [33].

Anoctamin-1 regulates chloride secretion in epithelia following elevation of [Ca^2+^]_in_ [34, 103]. Anoctamin-1 is expressed in the outer and inner retinal nuclear and ganglion cell layers, secretory epithelia (lung, pancreas, kidney) and sensory neurons and is activated by [Ca^2+^]_in_ [94]. Schreiber and Kunzelmann [35] first reported the expression of anoctamin-1 in human, porcine, bovine and mouse RPE cells where it was proposed to be the basolateral CaCC. Application of ATP to the apical surface of porcine RPE increased [Ca^2+^]_in_ and basolateral chloride conductance that was reduced in the presence of an anoctamin-1 inhibitor (niflumic acid). Thus, TMEM16A (anoctamin-1) is a significant basolateral CaCC in the RPE and can be activated by cell swelling to increase fluid transport to the choroid [35]. The intracellular regulation of calcium stores and the L-type Ca^2+^ channels by bestrophin provides a mechanism for the depolarization of the basolateral membrane and the light-rise of the EOG.

## Modulators of bestrophin

In the RPE, bestrophin-1 acts as a regulator of intracellular calcium stores primarily and mutations in bestrophin result in impaired calcium regulation in the RPE with consequent pathological changes including accumulation creating the hallmark vitelliform lesion [81, 86]. Bestrophin also conducts HCO_3_^−^ that if compromised may alter pH regulation in the RPE [104]. Bestrophin also regulates neurotransmitters with the glutamate metabolic enzyme (glutamic acid decarboxylases (GAD65)) increasing permeability to glutamate and GABA through bestrophin [105]. GABA also binds to bestrophin to increase chloride conductance which suggests the function of bestrophin may contribute to neuronal function [105]. A recent role for bestrophin in the central nervous system where it regulates the slow release of glutamate [106] and GABA from astrocytes [107] and has been proposed in the pathogenesis of Alzheimer’s Disease [108]. Thus, bestrophin-1 serves as a multifunctional channel protein whose disruption extends beyond simple chloride transport to encompass calcium homeostasis, pH regulation, and neurotransmitter release. The wide-ranging consequences of bestrophin dysfunction highlight its critical role in maintaining both retinal pigment epithelium integrity and broader neurological function.

## Candidates for the light-rise substance and receptor

Despite the first description of the clinical EOG by Arden et al. [11], the complete mechanistic pathway has remained unsolved with the identification of either the light-rise substance that is either released from the rod photoreceptors or RPE to initiate a rise in [Ca^2+^]_in_ through binding to or interacting with a light-rise receptor. The following section summarizes the main findings and proposes a possible mechanism with ATP as the light-rise substance. The establishment of chloride being the ion that led to basal membrane depolarization and the light-rise was understood through early experiments in retina-RPE-choroid preparations from gecko [38] and intact cat eye [39]. L-type Ca^2+^ channels were identified in rat RPE cells as the main calcium channel in the RPE [53] with a CaCC [109] important in the generation of the light-rise [110]. The understanding of calcium regulation through L-type Ca^2+^ channels, bestrophin, and TMEM16A/anoctamin-1 has helped explain the final stages of the light-rise with an increase in [Ca^2+^]_in_ and basolateral chloride conductance. However, the specific mechanism for the rise in [Ca^2+^]_in_ [110] is unclear with two possible contenders: Namely ATP and/or glutamate being the elusive light-rise substance [36]. Historically, Linsenmeier and Steinberg [39] proposed that the light-rise substance would diffuse from the neural retina and either bind to a receptor on the apical membrane of the RPE or diffuse into the RPE based on observations that the amplitude of the light-rise was dependent on stimulus area.

Several candidates and possible explanations for the light-rise substance or generation of the light-rise have been proposed and explored. The photoreceptor release of a Ca^2+^ dependent neurotransmitter such as dopamine was not supported by blockade of this mechanism by cobalt [111] and suppression of the light-rise by dopamine applied to the apical membrane of the RPE [112]. Further studies in chick found that the blockade of dopamine and 5-hydroxytryptamine synthesis led to a delay in the timing of the light peak amplitude but no change in the dark trough in chick eyes [113]. In contrast to the animal studies, the light-rise in Parkinson’s Disease is reduced in amplitude and the time to peak delayed [114] with the restoration of the light-rise by levodopa also supporting a possible role for dopamine signaling in the generation of the light-rise [115]. Thus, dopamine may modulate the light-rise but there is no direct evidence that dopamine is the light-rise substance.

The sympathomimetic catecholamine, epinephrine stimulates fluid transport in the RPE through an increased basolateral chloride conductance that increases the TEP [1] and follows an elevation of [Ca^2+^]_in_ [3]. Epinephrine levels in the rat retina also increased following light onset for two hours [116] which raised the possibility that epinephrine could be the light-rise substance as proposed by Joseph and Miller [117]. The epinephrine response was replicated in fetal RPE explants with an increase in the TEP when β (iso-protenerol) or an α_1_ (epinephrine) agonist was applied to the apical membrane with an increase in basolateral membrane conductance [69]. To date a direct pathway for the light triggered release of epinephrine to initiate the light-rise has not been demonstrated with the only clear evidence derived from the study of Hadjiconstantinou et al. [116] but the study did not show that light contributed to the relaese of epinephrine from the photoreceptors or other retinal cells. Thus epinephrine for now remains a possible but unlikely candidate until a direct link between light onset and direct photoreceptor release of epinephrine emerges.

Experiments with cAMP have been mixed with initial experiments in chick retina-RPE-choroid preparations showing a decrease in basolateral chloride conductance in the presence of cAMP [74]. However, in human fetal RPE cells cAMP increased basolateral chloride conductance via a 4,4′-diisothiocyanostilbene-2,2′-disulfonic acid (DIDS) sensitive CFTR channel [69]. However, the CFTR channel has been excluded as the generator of basolateral chloride conductance [118] given the LP:DT_ratio_ amplitude was unaffected in cystic fibrosis individuals [119]. DIDS suppressed to light-rise in chick which originally led to speculation that CFTR was involved in the light-rise [120], but DIDS also blocks anoctamin-1 which may have caused a similar effect of reducing basolateral chloride conductance, which was not known at the time [36].

One important possible mechanism for the light-rise was made by Mitchell [62] who found that RPE cell lines released ATP through apical CFTR channels and bind to apical P_2_Y_2_ purinergic receptors to elevate [Ca^2+^]_in_ suggesting ATP could be the substance responsible for initiating the light-rise [62, 71]. Supporting this model, ATP was known to elevate [Ca^2+^]_in_ in bovine RPE preparations [121]. The possible involvement of ATP is supported by other studies into retinal hypoxia and the effects on the light-rise. Linsenmeier et al. [122] used intact cat eyes to model the effects of gradual hypoxia and hypercapnia on the light peak following prior observations that when respiratory rate declined the light peak amplitude decreased slightly [39]. Hypoxia reduced the light peak in cat but had no effect on the ERG which highlighted that the light-rise was due to a change in the RPE’s physiological response to hypoxia rather than the retina. The outer retina in the dark consumes more oxygen than in the light [123, 124] with the pO_2_ at the level of the RPE, approximately 25 mmHg less in the dark than in the light. A speculation could be that the changes in pO_2_ would reduce the availability of ATP to drive the apical NaKATPase pump in the RPE and reduce the potential for ATP transport through CFTR to initiate the light-rise. However, if CFTR transport of ATP was responsible for autocrine signaling via purinergic receptors to trigger the light-rise then this model is not supported by normal light-rise response amplitudes in CF patients [119]. One solution may be that ATP release is via vesicular transport from the RPE as identified as an alternative pathway by Reigada and Mitchell (2005) [61] when the vesicular transport inhibitor, brefeldin A, basal and hypotonic induced ATP release from ARPE-19 cells was reduced. Vesicular transport of ATP may be a possible pathway for the autocrine stimulation of purinergic receptors with cell swelling shown to increase vesicular release of ATP in intestinal epithelial cell lines [125]. Could cell volume changes and retinal hypoxia underlie the release of ATP from RPE cells to initiate the light-rise and not a rod response? The possible role of glutamate that increases ATP release from the RPE through a mechanism that does not involve CFTR or vesicular transport is hard to reconcile [76] given high levels of glutamate in darkness would be expected to increase [Ca^2+^]_in_ and evoke a ‘light-rise’ response rather than the dark trough that is observed. Tentative clinical support for ATP as central to the generation of the light-rise comes from two patients (twin brothers) with a known mutation in the *COQ2* gene that encodes the coenzyme Q2 that is crucial component for the synthesis of ATP. In these two patients it was reported that the light-rise was absent while the full-field light and dark-adapted ERGs and pattern ERG were relatively preserved or within normal limits [126]. This finding most probably suggests that the ATP concentration within the RPE is important for the generation of the light-rise and that the origins of the light-rise depend upon ATP.

## Photoreceptor origin of the light-rise substance

Given that the rod photoreceptors were proposed to be the source of the light-rise substance as dark adaptation precedes the light-rise and that the spectral sensitivity of the light-rise was maximal at close to the peak absorption of rhodopsin as originally described by Arden and Kelsey [14]. However, other studies have shown a sensitivity to wavelengths of (472, 572 and 618 nm) implicating a possible cone contribution to the light-rise [127]. In a small study using participants with color blindness [128] the light-rise was evident with Blue-Green filters but not Red-Green filters and concluded that the light-rise was a mixed rod-cone response. A large study on the spectral sensitivity of the EOG light-rise demonstrated a peak sensitivity of 448 nm [129] rather than ~ 500 nm as Arden originally described [14]. In support of a photoreceptor origin of the light-rise, the G_α_ (transducin) knock-out mouse, that maintain normal retinal anatomy, the DC-ERG recorded light-rise was reduced in amplitude [130]. The reduced rate of phototransduction would in effect maintain the rods in a dark-adapted state with the cyclic nucleotide gated channels remaining open and activity of the photoreceptor NaKATPase pumps keeping the metabolic demand of the outer retina high with low ATP which may account for the lack of a light evoked RPE response if ATP is the light-rise substance.

A potential pathway where light might bypass the photoreceptors and trigger the light-rise directly through an interaction with the RPE has been proposed. Constable [131] postulated that light may trigger the release of IP_3_ from the lipids of the RPE to release intracellular calcium to gate open the CaCC which may have been linked to the shedding of rod outer segments at light onset and subsequent volume regulation through increasing basolateral chloride conductance. The possibility that melanopsin that is expressed in the RPE [132] was excluded as the possible light-rise receptor following evidence in a knock-out mouse model of the melanopsin *opn4*^*−/−*^ the light-rise was unaffected (Neal Peachey–personal communication).

The model for the initiation of the light-rise has rested on the belief that a ‘light rise substance’ is released from the rod photoreceptors and binds to an apical RPE receptor to increase intracellular calcium and ultimately an increase in basolateral chloride conductance [15, 140]. To question this model and view the damped oscillations of the standing potential in response to light as a physiological response of the RPE and not dependent on an external ‘light-rise’ substance to trigger the changes in [Ca^2+^]_in_ would be a more efficient mechanism. To that end the regulation of cell volume would be one possibility as the sub retinal space volume increases in light and fluid is transported through the RPE to the choroid. The slow damped oscillation of the EOG reflects the L-type Ca^2+^ channel activity in regulating [Ca^2+^]_in_ and sub retinal fluid transport. Calcium oscillations are mediated by L-type Calcium channels to regulate cell volume in the adrenal gland [141] and astrocytes [142] with a 3–4 min period and may account for the slow damped oscillation of the EOG while the RPE reduces sub-retinal fluid.

Whilst attention has focused on the light-rise of the EOG, any mechanism will also need to consider the dark through the fall in the standing potential during dark adaptation. It was proposed based on a mathematical modeling of the slow oscillations of the EOG that the dark and light oscillations have separate mechanisms based on the differences in the period and faster rate of dampening of the dark trough oscillations [133, 134]. The fall in the standing potential during dark adaptation implies a hyperpolarization of the basolateral membrane or a depolarization of the apical membrane or a decrease in total tissue resistance through a reduction in the tight junctions connecting the RPE cells [41]. The fall in the standing potential in the dark may also reflect the fall in availability of ATP. It is known that the dark trough shows high variability [134] that may reflect underlying physiological differences in RPE metabolism if ATP is implicated in this response. A similar result has also been reported in Kearns-Sayre syndrome affecting mitochondrial function. In two of the three cases the EOG was reported to be abnormal with normal and abnormal ERG findings reported [135].

## Non-photic effects on the standing potential

The light-rise of the EOG is naturally associated with light and its influence on the standing potential. However, changes in pO_2_ and pH can also influence the light-rise of the EOG suggesting, as Arden postulated a metabolic component to the generation of the light-rise given by observing acute drops in the standing potential during acute instance of retinal ischemia induced by pressing on the eye of a subject [14]. Later studies in isolated chick showed the light peak amplitude was reduced in hypoxic conditions before affecting the ERG which indicated the greater sensitivity of the RPE in hypoxic conditions that reduced basolateral chloride conductance [122]. Nakazaki et al. [136] using potassium selective micro-electrodes in chick RPE-retina-choroid preparations and with 20–30 min of hypoxia also found a fall in the TEP and increase in sub-retinal potassium concentration, due to a slowing of the photoreceptor and RPE apical membrane NaKATPase pumps [136]. Reductions in the standing potential have also been observed with hypercapnia, but not acidosis, in the artificially perfused intact cat eye preparation due to a fall in intracellular pH [137, 138]. Ethanol also produces a similar ‘alcohol-rise’ in dark adapted subjects [139]. Reductions in the standing potential have also been observed following intravenous infusions of either hypertonic [142] or bicarbonate solutions [143] but neither of these non-photic stimuli have developed into a clinical test of RPE function. The generation of an ‘ethanol rise’ suggests ethanol may also elevate [Ca^2+^]_in_ although the direct mechanism is unknown, but the ethanol-EOG is also reduced in RPE pathology and is likely to act directly on the RPE [144].

## Discussion

How to resolve the various studies that have examined the properties and origins of the light evoked and non photic responses of the RPE? Variability in clinical recordings throughout the day suggest an underlying circadian cycle to the EOG however, a circadian effect on the EOG has not been resolved with Anderson and Purple [145] reporting that the LP:DT_ratio_ was most stable and minimal at midday and exhibited a circadian rhythm across the 24-h recoding period. In contrast, Timmins and Marmor [134] recorded the EOG at one-hour intervals between 09:00 and 18:00 h and found higher absolute values of the LP and baseline DT in the morning than the evening-with no overall effect on the LP:DT_ratio_ during the daytime. Epinephrine [3, 69, 117], ATP [61, 62, 71, 72, 121, 146] and glutamate [76] increase [Ca^2+^]_in_ that could evoke the light-rise but which one of these candidates best explain the dark trough and light-rise’s slow oscillations? Retinal dopamine levels are high in light, but the dopaminergic neurons lie in the inner nuclear layer [147] and so photoreceptor release of dopamine may not be a direct sequela with leva-dopa restoring central dopamine levels that may explain the pharmacological response in Parkinson’s Disease. Currently there is no direct evidence for epinephrine release from photoreceptors, but light exposure does increase retinal epinephrine [148] although the time course for epinephrine increase has been demonstrated to be a rapid response that would fully account for the time course of the light-rise [116, 148]. Glutamate is an attractive candidate given the direct changes in glutamate linked to phototransduction, but glutamate release in the dark does not fit with the observed increase in the standing potential during the light phase and not the dark phase [76]. Schreiber and Kunzelmann [36] have proposed ATP and glutamate as a likely candidate for the light-rise substance, and a possible mechanism is outlined here based on ATP as the main modulator of [Ca^2+^]_in_ to gate open the anoctamin CaCC channels.

## ATP: the elusive light-rise substance?

The case for ATP is based on findings that show purinergic stimulation in HEK293 cell lines increases [Ca^2+^]_in_ and chloride currents in the presence of human Best1 [70]. Autocrine stimulation by ATP onto purinergic receptors through vesicular transport from the RPE cells also provides a pathway for ATP to elevate [Ca^2+^]_in_ [61, 71]. At an intracellular level, ATP is a critical regulator of bestrophin with the p.I201T mutation affecting the binding of ATP and bestrophin function [146]. Further links in support of ATP may be that ATP levels in the RPE decline with age that are associated with mitochondrial damage and levels of cytosolic [Ca^2+^]_in_ also decrease with age [149]. These are related decline in ATP and [Ca^2+^]_in_ and may account for the similar decline in the LP:DT_ratio_ with age, declining for approximately 0.13/decade [150].

**Fig. 3:**
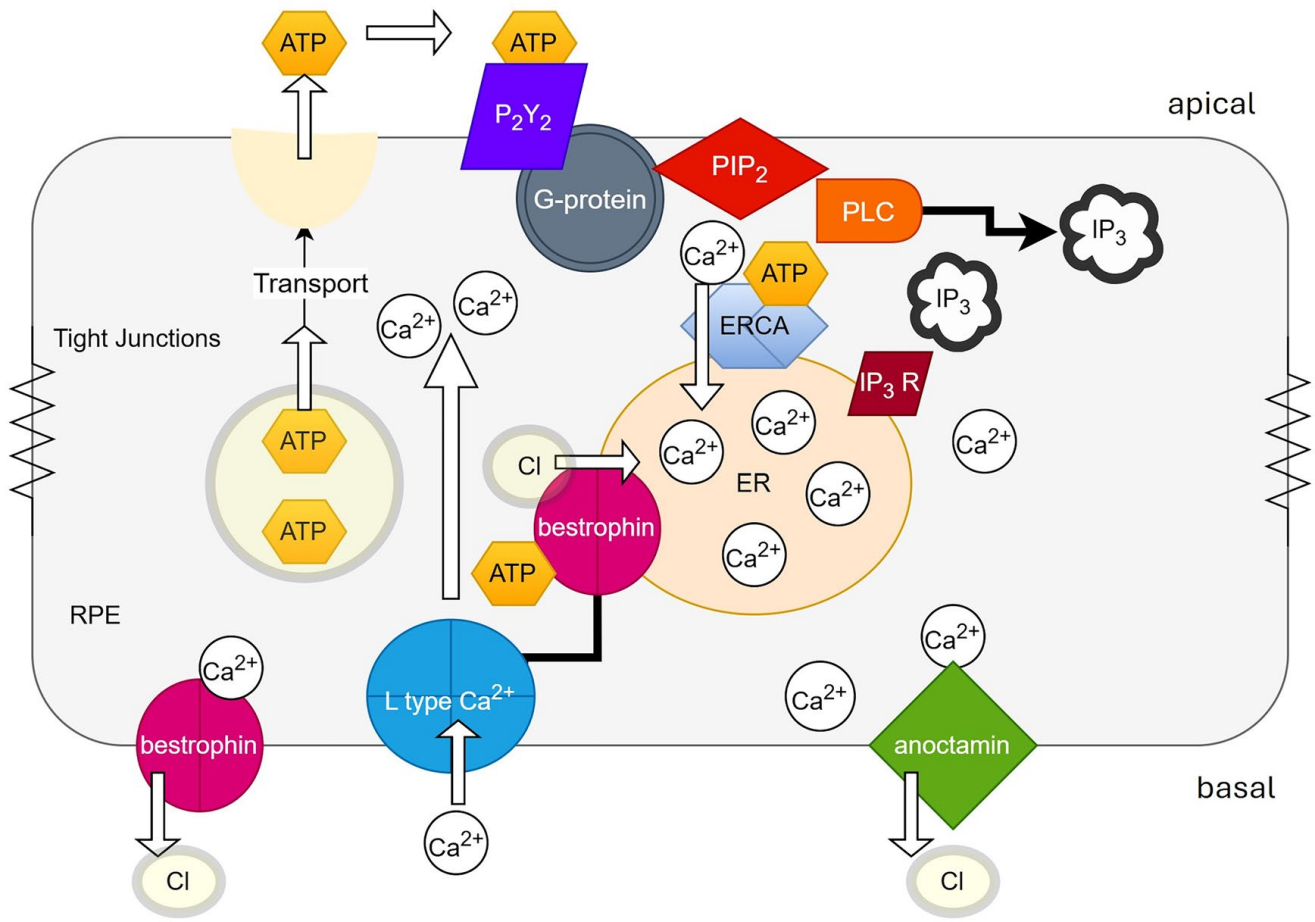
A possible mechanism of the light-rise of the EOG. In this proposed model, cell swelling and decreased metabolic rate enable the release of ATP via vesicular transport from the RPE. ATP binds to the apical purinergic P_2_Y_2_ G-protein coupled receptor. Conformational changes in the G-protein initiate the generation of the intracellular second messenger (IP_3_) through membrane bound phosphatidylinositol 4,5-bisphosphate (PIP_2_). IP_3_ triggers release of intracellular calcium stores from the endoplasmic reticulum which then gates open the basolateral calcium activated chloride channel anoctamin which depolarizes the basolateral membrane. This increases the trans-epithelial potential as well as the standing potential that is recorded clinically as the light-rise. The role of bestrophin is central to the re-uptake of cytosolic calcium for transport into the endoplasmic reticulum via the active endoplasmic reticulum calcium ATPase pump. Bestrophin links to and regulates calcium entry via the L-type calcium channel and transports chloride into the endoplasmic reticulum to increase the electrochemical driving force for calcium entry into the endoplasmic reticulum. In this model the dark trough is the reversal of the biochemical pathway with a decrease in ATP and cell volume linked to an increased metabolic demand of the RPE to regulate the ions of the sub-retinal space. At this stage the identity of ATP as ‘the’ light-rise substance, and this possible pathway is yet to be verified experimentally

With changes in ATP consumption in the outer retina being approximately four-fold in the dark, compared to the light reflecting increased activity of the outer segment NaKATPase pumps [151]. ATP and adenosine are also recognized to play an important role in communication between the RPE and the photoreceptors that increase fluid transport [121]. There is evidence for cross-talk between adenosine and ATP with the application of adenosine potentiating the effect of ATP to elevate [Ca^2+^]_in_ in cultured human RPE cells [72]. One of the main functions of the RPE is to regulate fluid within the subretinal space that follows light onset [152, 153]. With evidence for ATP transport via vesicular release in RPE and epithelial cells [61, 125] then cell swelling may release ATP, combined with the reduced metabolic demand of the outer retina in light compared to dark. This would increase the availability of ATP to either be released by the RPE or to directly bind with bestrophin [61, 146] which also is an important regulator of cell volume [154]. The fall in ATP in the dark may contribute to the dark trough, with a fall in the standing potential caused by reduced basolateral chloride conductance, but this aspect of the EOG has not been directly studied to date. Figure 3 provides a possible pathway and mechanism of the EOG based on the function of bestrophin with ATP acting as the light-rise substance.

The EOG is typically abnormal in the presence of marked retinal degeneration such as retinitis pigmentosa that supports a photoreceptor origin of the light-rise [155]. However, in early-to-moderate photoreceptor degeneration, the EOG light-rise can remain relatively preserved with 59% of X-linked retinitis pigmentosa carriers maintaining a normal EOG [156]. In mouse retinal degeneration models of Prph2^Rd2/+^ revealed that while the ERG a-wave declined in parallel with photoreceptor loss, the DC-ERG light-peak and c-wave did not follow the same linear relationship between loss of function and outer segment structure supporting a non-photoreceptor dependence on the light-rise. RPE responses showed relative preservation beyond what would be predicted by photoreceptor loss alone, demonstrating a non-proportional relationship between photoreceptor input and RPE function [157]. Thus the light rise can occur in the presence of photoreceptor dysfunction.

## Conclusions

The complete story of the EOG is yet to be fully explained. ATP is a likely candidate for initiating the rise in [Ca^2+^]_in_ and bestrophin interacting with the L-type Ca^2+^channel to regulate endoplasmic stores with anoctamin providing the basolateral CaCC and depolarization if the RPE basolateral membrane. Therefore, the EOG test could be viewed as a test of RPE metabolic health primarily and not a specific test of rod-RPE integrity as generally viewed. Naturally the EOG also involves bestrophin and chloride transport, but the availability of ATP is also important for the generation of the light-rise. In contrast to Schreiber and Kunzelman’s proposal that ATP or glutamate are released by photoreceptors as the light-rise substance [36], the contention proposed is that the source of the ATP is inherent to the RPE and fluctuates with metabolic demand and fluid regulation between light and dark.

Further questions remain about the possible role of glutamate and potential cross talk with NMDA, adenosine and purinergic receptors while cross-talk may provide the mechanism to explain the damped oscillation and regulation of [Ca^2+^]_in_ over time. Further clinical studies in patients with mutations affecting ATP such as COQ2 and mitochondrial disorders where studies tend to focus on the ERG rather than EOG findings in conditions such as Leber’s Hereditary Optic Neuropathy or Kearns-Sayre syndrome would be welcomed to further characterize the role of ATP in the light-rise.

## Data Availability

No datasets were generated or analysed during the current study.
